# Supplementary figures and images for: Acetylation of N-terminus and two internal amino acids is dispensable for degradation of a protein that aberrantly engages the endoplasmic reticulum translocon
Source: PeerJ. 2017 Aug 22;5:e3728. doi: 10.7717/peerj.3728 (PMC5571791; doi:10.7717/peerj.3728)

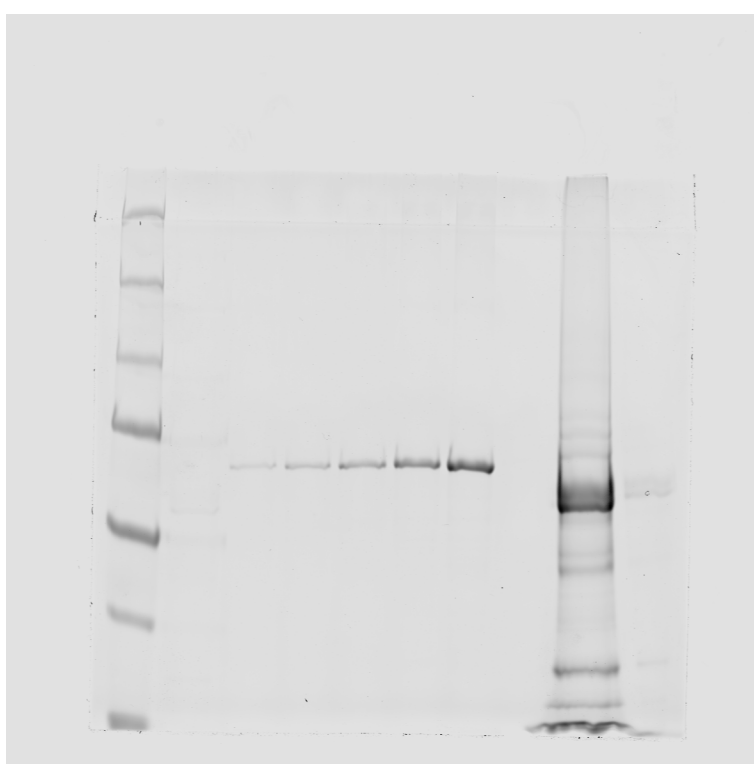

Supplement: Supplemental Information 1 [file peerj-05-3728-s001.pdf]

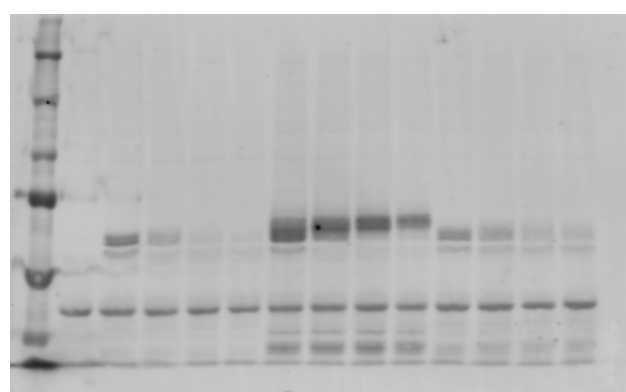

Supplement: Supplemental Information 2 [file peerj-05-3728-s002.pdf]

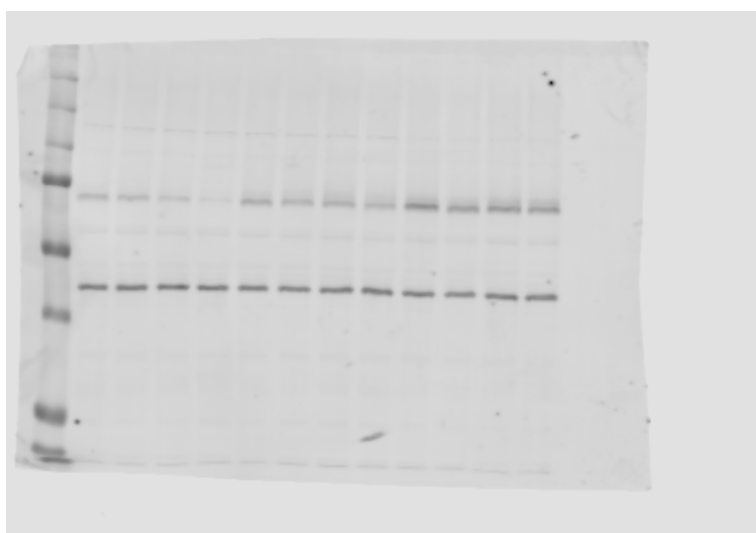

Supplement: Supplemental Information 3 [file peerj-05-3728-s003.pdf]

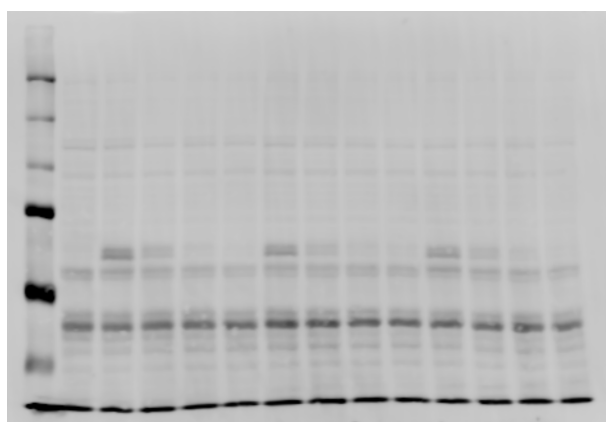

Supplement: Supplemental Information 4 [file peerj-05-3728-s004.pdf]

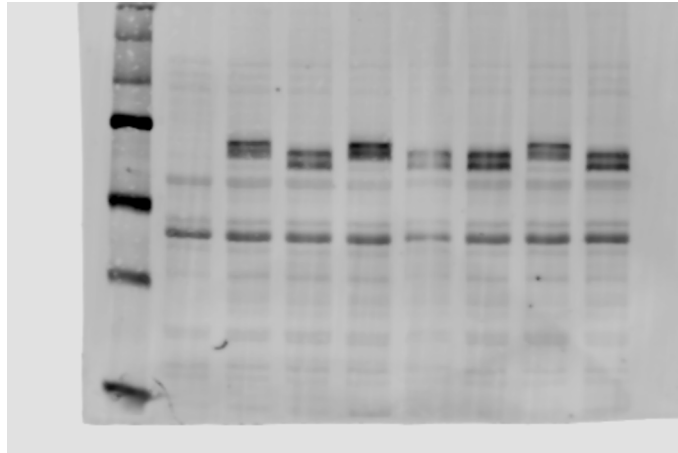

Supplement: Supplemental Information 5 [file peerj-05-3728-s005.pdf]

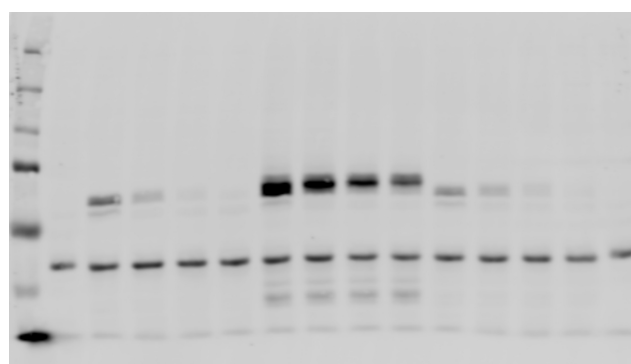

Supplement: Supplemental Information 6 [file peerj-05-3728-s006.pdf]

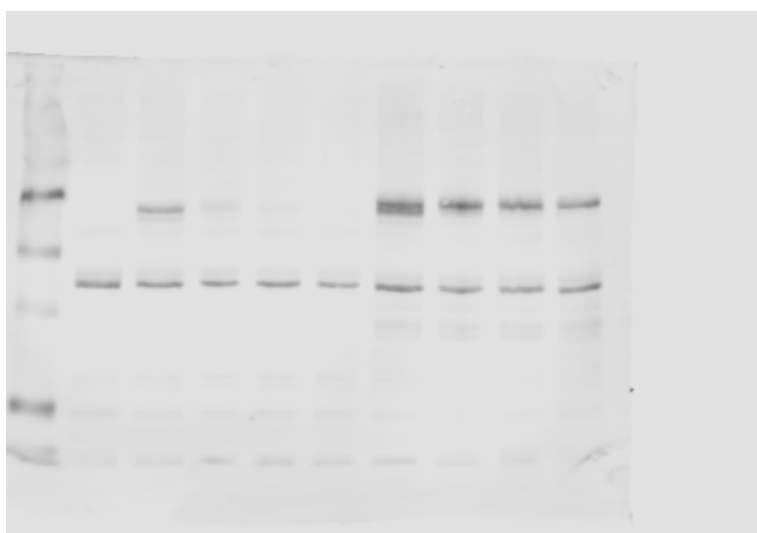

Supplement: Supplemental Information 7 [file peerj-05-3728-s007.pdf]
